# Multifunctional properties of *Lactobacillus plantarum* strains WiKim83 and WiKim87 as a starter culture for fermented food

**DOI:** 10.1002/fsn3.1075

**Published:** 2019-07-02

**Authors:** Ji‐Hye Jung, Su‐Ji Kim, Jae Yong Lee, So‐Ra Yoon, Su‐Yeon You, Sung Hyun Kim

**Affiliations:** ^1^ Hygienic Safety and Analysis Center World Institute of Kimchi Gwangju Korea

**Keywords:** antimicrobial activity, antioxidant activity, *Lactobacillus plantarum*, probiotic, *β*‐galactosidase activity

## Abstract

This study aimed to evaluate the safety (hemolysis and enzyme activity), probiotic properties (gastrointestinal tract tolerance, adhesion, hydrophobicity, and auto‐aggregation), and functional characteristics (antimicrobial, antioxidant, and *β*‐galactosidase activities) of lactic acid bacteria (LAB), isolated from kimchi, in order to select a multifunctional LAB strain for starter culture in fermented food. The five isolated strains included *Lactobacillus plantarum* WiKim83, *L*.* plantarum* WiKim84, *Pediococcus pentosaceus* WiKim85, *P*.* pentosaceus* WiKim86, and *L*.* plantarum* WiKim87, as identified by 16S rRNA gene sequence analysis; they were confirmed to be nonhemolytic and not able to produce *β*‐glucuronidase, a carcinogenic enzyme. Probiotic properties of the five LAB strains were evaluated relative to those of commercial *Lactobacillus rhamnosus* GG, and results revealed probiotic potential of three strains (*L*.* plantarum* WiKim83, *L*.* plantarum* WiKim84, and *L*.* plantarum* WiKim87) to be superior. *L*.* plantarum* WiKim84 showed high antimicrobial activity against pathogens, and *L*.* plantarum* WiKim83 exhibited the highest antioxidant and *β*‐galactosidase activities. Based on the probiotic and functional properties, the main characteristics of each strain were highlighted and two of them, *L*.* plantarum* WiKim83 and *L*.* plantarum* WiKim87, were selected as the most potent by principal component analysis. These strains showed antimicrobial, *β*‐galactosidase, and antioxidant activities, which recommend their suitability as starter culture in various fermented foods.

## 1 INTRODUCTION

Kimchi is a typical Korean side dish, prepared by fermenting kimchi cabbage and other ingredients such as radish, garlic, seafood, and red pepper powder; it is recognized as a functional food owing to its content of a variety of biochemical substances (Jang, Chung, Yang, Kim, & Kwon, 2015). Additionally, it is a source of probiotic bacteria, namely *Lactococcus*, *Lactobacillus*, *Pediococcus*, and *Leuconostoc* (Jung et al., 2011).

Starter culture strains are often used to improve the sensory characteristics of fermented foods, maintain safety and quality, and promote nutrition (Champagne & Møllgaard, 2008). In recent years, exploring microorganisms with various functions, for the starter culture, has been a major trend in food microbiology (Perricone, Bevilacqua, Corbo, & Sinigaglia, 2014). Several studies have been reported on food products using multifunctional microorganisms (Holko, Hrabě, Šalaková, & Rada, 2013; Lavermicocca et al., 2005). Especially, lactic acid bacteria (LAB) are strongly recommended for starter culture in fermentation due to its advantages regarding probiotic characteristics, antimicrobial production, beneficial enzyme production, and enhancement of functionality (Di Cagno, Coda, Angelis, & Gobbetti, 2013; Randazzo et al., 2013).

Lactic acid bacteria are commonly considered safe and are widely used as starter culture in the production of fermented foods (Carr, Chill, & Maida, 2002). LAB are potent as probiotics and exhibit beneficial effects such as antimicrobial production, beneficial enzyme production, immune regulation, and antioxidant activity (Gerritsen, Smidt, Rijkers, & Vos, 2011).

The microbiota in the intestine has significant influence on host immunity, nutrition, and physiological function (O'Sullivan et al., 2005). Probiotics are defined as “live microorganisms, which upon ingestion in adequate amounts, confer a health benefit to the host” (FAO/WHO, 2006). They are safe for the host when consumed, and their therapeutic role is enhanced due to the improvement of intestinal microbial communities and their subsequent correlation with human physiology and disease pathogenesis (O'Sullivan et al., 2005).

The FAO/WHO guidelines for assessing probiotics recommend conducting in vivo experiments on strains that have demonstrated potential health benefits based on in vitro experiments (FAO/WHO, 2006). The criteria for the main selection of probiotics based on the FAO/WHO guidelines are safety (nonpathogenic strains without toxicity), resistance to gastric and bile acids, epithelial cell adhesion, and antimicrobial activity (competition against pathogens). Furthermore, since probiotic properties are strain‐specific, each strain characteristic needs to be tested (Monteagudo‐Mera et al., 2012). Therefore, in this study, probiotic strains were selected based on the selection criteria of the FAO/WHO guidelines and previous research methods.

Many research groups have studied the functional characteristics and suitability of the starter culture of LAB as a probiotic (Lee et al., 2016; Nguyen et al., 2013; Son et al., 2017). However, LAB with different functional combinations need to be developed for use as the starter culture in various kinds of fermented foods. In this study, different strains of LAB were isolated from kimchi, and safety, probiotic properties, antimicrobial activity against ten food pathogens, *β*‐galactosidase activity, and antioxidant function were evaluated.

## 2 MATERIALS AND METHODS

### 2.1 Bacterial strains and culture

*Lactobacillus rhamnosus* GG (LGG, ATCC 53103), a commercial probiotic strain (control) was cultured in Man–Rogosa–Sharpe (MRS; Oxoid Ltd.) broth (pH 6.5) at 37°C for 24 hr. *E*.* coli* O157: H7 ATCC 43895, *Bacillus cereus* ATCC 10876, *Staphylococcus aureus* ATCC 25923, *Listeria monocytogenes* ATCC 13932, *Salmonella enterica* subsp. *enterica* serovar Typhimurium ATCC 13311, *Escherichia coli* ATCC 35218, *Vibrio parahaemolyticus* ATCC 17802, and *Yersinia enterocolitica* ATCC 23715 were aerobically propagated in tryptic soy broth (TSB; Difco) at 37°C for 24 hr. *Clostridium perfringens* ATCC 13124 was anaerobic cultured in cooked meat medium (Difco) at 37°C for 24 hr. *Campylobacter jejuni* ATCC 33291 and *Campylobacter coli* ATCC 43478 were cultured in Mueller Hinton (Difco) media at 42°C for 48 hr under microaerobic conditions. ATCC strains were purchased from the American Type Culture Collection.

### 2.2 Isolation and selection of LAB

Lactic acid bacteria were isolated from kimchi prepared in a South Korean temple. Kimchi samples were mixed with a hand blender, filtered through sterilized gauze, spread on MRS agar medium with 2% CaCO_3_, and incubated at 37°C for 48 hr. Among the cultivated strains on the MRS agar plate, those forming clear zones around them, owing to acid production, were isolated. The well‐diffusion assay (Fontana, Cocconcelli, Vignolo, & Saavedra, 2015) was used to determine the antimicrobial activity of the isolated strains. Cultured pathogens (*S*.* aureus*, *L*.* monocytogenes*, and *E*.* coli*) were plated on TSB agar plates at 10^6 ^CFU/ml. The plates were allowed to dry, and a sterile cork borer 7 mm in diameter was used to cut uniform wells in the agar. The isolated strains were cultured in MRS medium at 37°C for 24 hr and were prepared at a concentration of approximately 10^9^ CFU/ml and used to fill each well. After incubation at 37°C for 24 hr, the plates were observed for the zone of inhibition around the well. Among the isolated strains, those with excellent antimicrobial activity were selected and used for this study. All selected LAB strains were preserved at −80°C as 25% (v/v) glycerol stocks. The strains were cultured twice before subsequent experiments.

### 2.3 Identification of LAB

The five selected LAB strains were identified according to their 16S rRNA gene sequences using a kit (Macrogen), following the manufacturer's instructions, and an ABI prism 3730XL DNA analyzer (Applied Biosystems). The sequences were compared with those in GenBank database using the BLASTN program (http://blast.ncbi.nlm.nih.gov/Blast.cgi).

### 2.4 Hemolytic activity

Hemolytic activity was confirmed by streaking LAB on a 7% sheep blood agar base and incubating for 24 hr at 37°C. Hemolysis was determined by the formation of clear zones around the colonies (*β*‐hemolysis, clear zones; *γ*‐hemolysis, no zone). *E*.* coli* O157: H7, which exhibits *β*‐hemolysis activity, was used as a positive control.

### 2.5 Enzyme production

Enzyme production by the isolated strains was assessed with an API ZYM kit (BioMérieux). Cultures of each strain were centrifuged (13,000 × *g* for 15 min at 4°C), and the pellets (10^5^ CFU/ml) were added to individual cupules by reattachment to sterilized saline solution containing 0.85% sodium chloride. After 4‐hr inoculation at 37°C, reagents ZYM A and ZYM B were consecutively supplemented to the cupules, followed by observation of color change after a 5‐min reaction in a bright place. The progress of substrate hydrolysis (nmol of product) was decided based on the extent of color change.

### 2.6 Probiotic properties

#### 2.6.1 Survival under gastrointestinal tract (GIT) conditions

The ability of the isolated strains to tolerate acid, gastric juice (GJ), and bile salt (BS) was determined as described in a previous study (Oh & Jung, 2015), with minor modifications. Approximately 9 log CFU/ml cells were re‐suspended in acid (1 N HCl buffer, pH 2.5) or GJ (3 mg of pepsin in 1 ml of 0.5% saline buffer, pH 2.5) or BS (0.3% oxgall in PBS, pH 7.4). Cells were cultured at 37°C for 2 hr in acid or GJ, and for 24 hr in BS, and re‐suspended in MRS broth after incubation. After a 24‐hr incubation at 37°C in MRS agar, the live cells were counted. Comparisons were performed to control samples, where the cells were suspended in MRS media in the absence of acid, GJ, or BS. LGG, a commercial probiotic strain, was used as a positive control.

#### 2.6.2 Adhesion to Caco‐2 cells

The adhesion of isolated strains was evaluated using methods that were slightly modified from those of previous studies (Lee et al., 2015). Caco‐2 cells (KCLB 30037.1) were cultured in Dulbecco's modified Eagle's medium (DMEM; Hyclone), supplemented with 10% fetal bovine serum (FBS; Hyclone), 1% 10,000 IU/ml streptomycin/penicillin (Hyclone), 1% nonessential amino acids (Hyclone), 10 mM HEPES (Hyclone), 1 mM sodium pyruvate (Hyclone), and 1 mM _L_‐glutamine (Hyclone), at 37°C in a 5% CO_2 _atmosphere. To measure the rate of adhesion, the cell monolayer was seeded at a concentration of 10^5^ cells/ml in 24‐well plates, and strains were added at approximately 10^9^ CFU/ml in DMEM, without antibiotics and FBS, and cultured for 2 hr at 37°C with 5% CO_2_. Caco‐2 monolayers were then washed three times with PBS to remove the unattached LAB, after which 1 ml of 0.05% (v/v) triton X‐100 (Sigma‐Aldrich) was added to each well to detach the cells. Serially diluted cell suspensions were then plated on MRS agar and cultured at 37°C for 24 hr to determine the percentage of viable bacteria. LGG was used as a positive control.

#### 2.6.3 Hydrophobicity and auto‐aggregation

Hydrophobicity and auto‐aggregation of the strains were measured in accordance with previous methods (Ren et al., 2014), with minor modifications. The isolated strains and LGG were incubated in MRS broth (37°C for 24 hr), and the cultures were harvested thereafter. The cells were washed twice with PBS and then diluted to an optical density of 0.5 ± 0.02 (A0) at 600 nm. For the hydrophobicity assay, a 3 ml cell suspension was mixed with 1 ml of chloroform and cultured at 25°C for 30 min to separate the aqueous layer from the chloroform layer. The aqueous phase was identified based on the OD_600_ value (A1), and hydrophobicity (%) was calculated from the following equation:

$$\text{Hydrophobicity}\left(\%\right)=\left(\text{A}0-\text{A}1\right)/\text{A}0\times 100.$$

For auto‐aggregation, a 10 ml cell suspension was cultured at 25°C for 24 hr, and the OD_600_ of the solution (based on a 1 ml aliquot) was recorded (A1). Auto‐aggregation (%) was expressed based on the following equation:

$$\text{Auto-aggregation}\;\left(\%\right)=\left(\text{A}0-\text{A}1\right)/\text{A}0\times 100.$$

### 2.7 Antimicrobial activities against foodborne pathogens

The antimicrobial activity of strains against 10 foodborne pathogens was evaluated by well‐diffusion assays. The isolated strains were incubated at 37°C for 24 hr in MRS medium, following which cultures were centrifuged (13,000 × *g* for 15 min at 4°C), and the supernatant was drawn into an injector and passed through a filter with a 0.45‐µm pore diameter (Millipore). The indicator microorganisms used to assess the antimicrobial activity were *B*.* cereus*, *S*.* aureus*, *L*.* monocytogenes*, *C*.* perfringens*, *S*. Typhimurium, *E*.* coli*, *V*.* parahaemolyticus*, *Y*.* enterocolitica*, *C*.* jejuni*, and *C*.* coli*. Each pathogenic strain was spread on TSB agar plates at a concentration of 10^6 ^CFU/ml. Wells (7 mm in diameter) were cut, and the supernatant was filtered; the optimal culture conditions of each pathogen were used in each well for 24–48 hr. The antibiotic gentamicin (at 30 μg/ml) was used as a positive control. The diameters of the inhibition zones around each well were determined using calipers.

### 2.8 Antioxidant activity

#### 2.8.1 2,2‐diphenyl‐1‐picrylhydrazyl (DPPH) radical‐scavenging

DPPH radical‐scavenging activity (DPPH^+^) was evaluated using the culture supernatant from LAB strains, in accordance with a previously described method (Riaz Rajoka et al., 2017), with some modifications. The 0.2 ml culture supernatant was mixed with 2.8 ml of DPPH solution (60 µM), and the reaction was allowed to proceed in the dark for 30 min at 25°C. The reaction solution was centrifuged, and the absorbance at 517 nm was recorded by an ELISA reader (Amersham Biosciences). Ascorbic acid was used as the standard material, and culture medium at the same ratio as the culture supernatant was used as a control. DPPH^+^ (%) was calculated according to the following equation:

$${\text{DPPH}}^{+}\left(\%\right)=\left[1-\left({\text{A}}_{\text{sample}}/{\text{A}}_{\text{control}}\right)\right]\times 100.$$

#### 2.8.2 2,2′‐azinobis (3‐ethylbenzothiazoline‐6‐sulfonic acid) (ABTS) cation radical‐scavenging

ABTS radical cation‐scavenging activity (ABTS^•+^) was measured based on the method of Pieniz, Andreazza, Anghinoni, Camargo, and Brandelli (2014) with some modifications. The ABTS radical cation solution was prepared by allowing the ABTS solution to react with 2.45 mM potassium persulfate in the dark at 25°C for 16 hr. The ABTS solution was diluted with ultrapure water to an OD_734 _of 0.7_,_ with ascorbic acid as the standard control. The culture supernatant and diluted ABTS solution were mixed together, and the OD_734_ was recorded at 30‐s intervals for 5 min using an ELISA plate reader. The standard material and control were the same as those used for DPPH^+^. ABTS^•+ ^was calculated using the same equation as that for DPPH^+^.

#### 2.8.3 *β*‐Carotene

*β*‐Carotene bleaching activity was measured using a method slightly modified from that of Kachouri et al. (2015). Briefly, 0.4 mM *β*‐carotene solution was prepared using 0.2 mg *β*‐carotene, 40 μl linoleic acid, 400 μl tween 80, and 10 ml chloroform; the chloroform was subsequently removed by a vacuum distiller at 50°C in a rotary evaporator. The mixture was poured into 100 ml of distilled water to prepare an emulsion. To 4 ml of the emulsion, 0.2 ml culture supernatant (PBS, control) was added and incubated at 50°C for 2 hr; absorbance at 470 nm was subsequently recorded. The standard material and control were the same as those used for DPPH^+^. *β*‐Carotene activity was calculated according to the following equation:

$$\begin{array}{c} \beta \text{-Carotene}\;(\%)\\ =\left[\left({\text{A}}_{\text{sample,}\;\text{2}\;\text{hr}}-{\text{A}}_{\text{control,}\;\text{2}\;\text{hr}}\right)/\left({\text{A}}_{\text{control,}\;\text{0}\;\text{hr}}-{\text{A}}_{\text{control,}\;\text{2}\;\text{hr}}\right)\right]\times 100. \end{array}$$

### 2.9 *β*‐Galactosidase

*β*‐Galactosidase assay was performed by a method slightly modified from that reported previously (Vidhyasagar & Jeevaratnam, 2013). LAB were cultured in MRS medium for 24 hr, centrifuged, and washed thrice with sterilized PBS. Thereafter, 900 μl of P buffer (60 mM Na_2_HPO_4_, 40 mM NaH_2_PO_4_, and 50 mM *β*‐mercaptoethanol, pH 7.2), 0.1 ml of chloroform (Sigma‐Aldrich), and 0.05 ml of SDS (Sigma‐Aldrich) were mixed with 100 μl of the cell pellet (re‐suspended in P buffer), and absorbance of the cells at 560 nm was recorded. The enzyme assay was conducted by mixing the cell suspension (900 μl) with 0.2 ml of P buffer, supplemented with 4 mg/ml *o*‐nitrophenyl‐*β*‐D‐galactopyranoside (Sigma‐Aldrich). The reaction was then quenched by the addition of 0.3 ml of 1 M sodium carbonate (Sigma‐Aldrich). The reaction mixture was centrifuged, absorbance at 420 and 550 nm was recorded, and *β*‐galactosidase activity was calculated by the following equation:

$$\begin{array}{c} \beta \text{-Galactosidase activity}\;\left(\text{Miller units},\;\text{MU}\right)\\ =\left[{\text{A}}_{420}-\left(1.75\times {\text{A}}_{550}\right)/15\;\min\;\times \;1\;\text{ml}\times {\text{A}}_{550}\right]\times 100. \end{array}$$

### 2.10 Statistical analysis

The displayed results are expressed as the mean and standard deviation (*SD*) of three replicates. Statistical analysis was performed using SPSS software (v23.0 for Windows; IBM Corp.). The data were subjected to a *t* test and analysis of variance, the mean value was separated using Duncan's multiple‐range test, and statistical significance was defined as *p* < 0.05. Data regarding the probiotic properties and functional characteristics of the isolated LAB strains were subjected to principal component analysis (PCA) using Pearson's correlation and XLSTAT software (18.06; https://www.xlstat.com/en/).

## 3 RESULTS AND DISCUSSION

### 3.1 Isolation, selection, and identification of LAB

The LAB strain (*n* = 30) that produced acid in 2% CaCO_3_ MRS agar medium was isolated from the temple kimchi samples. Ten strains with different morphological characteristics among those isolated were first selected (data not shown), and their antimicrobial activities against three pathogenic strains were confirmed (Table S1). As a result, five strains (WiKim83, WiKim84, WiKim85, WiKim86, and WiKim87) with excellent antimicrobial activity were selected for further experiments. The five LAB strains were identified as *L*.* plantarum* WiKim83, *L*.* plantarum* WiKim84, *Pediococcus pentosaceus* WiKim85, *P*.* pentosaceus* WiKim86, and *L*.* plantarum* WiKim87 by 16S rRNA gene sequencing (Table 1).

**Table 1:** Molecular identification of selected lactic acid bacteria strains by 16S rRNA gene sequencing

Strain	Molecular identification	Similarity (%)	Accession number^a^
WiKim83	*Lactobacillus plantarum*	99.9	MH707244
WiKim84	*L*.* plantarum*	99.9	MH707245
WiKim85	*Pediococcus pentosaceus*	99.9	MK544837
WiKim86	*P*.* pentosaceus*	99.9	MK552379
WiKim87	*L*.* plantarum*	99.9	MH707246

a GenBank database.

### 3.2 Safety evaluation of LAB

We confirmed that the five isolated LAB strains exhibited nonhemolytic activities (Table 2), whereas the positive control *E*.* coli* O157: H7 exhibited *β*‐hemolysis (data not shown). The determination of hemolytic activity represents a safety evaluation to select probiotic strains (FAO/WHO, 2006). None of the five LAB strains produced enzymes like *β*‐glucuronidase, which are implicated in carcinogenicity and the induction of mutations (Table 2). Since the food industry requires careful assessment of the safety and usefulness of the strains prior to their use in food (Parvez, Malik, Ah Kang, & Kim, 2006), our results clearly indicate suitability of the five LAB strains in terms of safety and utility for use as probiotics.

**Table 2:** Hemolysis and enzyme production by the isolated lactic acid bacteria strains

	*L*.* plantarum* WiKim83	*L*.* plantarum* WiKim84	*P*.* pentosaceus* WiKim85	*P*.* pentosaceus* WiKim86	*L*.* plantarum* WiKim87
Hemolysis	*γ*	*γ*	*γ*	*γ*	*γ*
Enzyme^a^					
Control	0	0	0	0	0
Alkaline phosphatase	0	0	0	10	0
Esterase	0	0	0	5	0
Esterase lipase	0	0	0	5	0
Lipase	0	0	0	10	0
Leucine arylamidase	20	20	≥40	≥40	30
Valine arylamidase	0	0	20	30	20
Cystine arylamidase	0	0	0	10	0
Trypsin	0	0	0	10	0
α‐Chymotrypsin	0	0	0	0	0
Acid phosphatase	0	0	0	30	0
Naphthol‐AS‐BI‐phosphohydrolase	0	0	20	30	0
α‐Galactosidase	0	0	0	20	0
β‐Galactosidase	20	20	20	30	≥40
β‐Glucuronidase	0	0	0	0	0
α‐Glucosidase	20	20	0	10	20
β‐Glucosidase	20	20	20	20	20
N‐Acetyl‐β‐glucosaminidase	20	20	30	5	30
α‐Mannosidase	0	0	0	0	0
α‐Fucosidase	0	0	0	0	0

a Amount of enzymes derived from isolated LAB strains according to the API ZYM kit. All values are in nmol.

### 3.3 Probiotic properties of LAB

#### 3.3.1 Tolerance of GIT conditions

The ability to tolerate GS and BS in the upper GIT is an important requirement for potential probiotics (Gueimonde & Salminen, 2006). Therefore, tolerance to acid (1 N HCl [pH 2.5]), GJ (3 mg/ml pepsin [pH 2.5]), and BS (0.3% [pH 7.4]) was assessed for each strain. Results revealed that tolerance of LAB to GIT was significantly different across strains (Table 3). Among the LAB strains examined, *L*.* plantarum* WiKim83, *L*.* plantarum* WiKim84, and *L*.* plantarum* WiKim87 displayed high tolerance to acid, GJ, and 0.3% BS. In particular, the survival of the three *L*.* plantarum* strains was greater than 91%, which was similar to or higher than that of LGG (*p* < 0.05). Most LAB maintained high viability in GS at pH ≥3, but had low viability at pH ≤2.5. In addition, the tolerance of LAB to BS also varied across strains; *L*. *plantarum* and *L*. *fermentum* were previously reported to be highly resistant to BS (Huang et al., 2015; Tulumoglu, Kaya, & Simsek, 2014). *L*.* plantarum* is already known for its resistance to extreme GIT and is used as a starter culture for fermented foods and probiotics. In particular, *L*.* plantarum* isolated from fermented foods has been reported to have high resistance. *L*.* plantarum* isolated from Korean kimchi was found to survive at pH 2.5 for 2 hr (Lee et al., 2011), and *L*.* plantarum* isolated from yogurt was maintained at 5 log CFU/ml after 4‐hr incubation at pH 1.5 (Chen et al., 2014). Further, *L*.* plantarum* ZDY 2013 isolated from Chinese traditional acid beans survived at pH 2.0 for 6 hr and 65.84% of bacteria survived upon GIT simulation (Huang et al., 2015). In contrast, nine *L*.* plantarum* strains isolated from cheese were reduced to 6 log CFU/ml after incubation with GIT, from an initial inoculum of 9 log CFU/ml (Zago et al., 2011). *L*.* plantarum* WLPL04 had a reduced survival rate, <2 log CFU/ml after incubation in 0.3% BS for 24 hr (Jiang et al., 2016). The fact that the three *L*.* plantarum* strains tested here, WiKim83, WiKim84, and WiKim87, had high survival in GIT suggest that they could be orally administered as probiotics.

**Table 3:** In vitro probiotic properties of isolated lactic acid bacteria strains

Strains	Gastrointestinal tract tolerance (log CFU/ml)				Adhesion (%)	Hydrophobicity (%)	Auto‐aggregation (%)
	Initial	Acid	Gastric juice	Bile salt			
*L*.* plantarum* WiKim83	9.15 ± 0.05	9.16 ± 0.06^a^	8.93 ± 0.26^a^	9.20 ± 0.15^a^	77.33 ± 2.08^a^	50.61 ± 0.76^a^	93.28 ± 0.01^a^
*L*.* plantarum* WiKim84	9.17 ± 0.06	8.27 ± 0.11^b^	8.06 ± 0.32^b^	9.15 ± 0.60^a^	71.00 ± 2.00^bc^	37.70 ± 0.42^c^	90.50 ± 4.75^a^
*P*.* pentosaceus* WiKim85	9.14 ± 0.07	6.01 ± 0.43^c^	5.49 ± 0.32^c^	6.75 ± 0.14^b^	54.67 ± 3.51^d^	11.76 ± 2.11^d^	93.39 ± 0.25^a^
*P*.* pentosaceus* WiKim86	9.22 ± 0.98	5.63 ± 0.21^c^	5.35 ± 0.13^c^	5.84 ± 0.35^c^	49.33 ± 2.08^e^	48.22 ± 1.27^ab^	85.04 ± 0.08^b^
*L*.* plantarum* WiKim87	9.13 ± 0.06	9.16 ± 0.04^a^	8.98 ± 0.07^a^	9.20 ± 0.52^a^	73.05 ± 1.53^ab^	44.65 ± 1.52^b^	91.48 ± 0.72^a^
*L*.* rhamnosus* GG	9.14 ± 0.35	8.26 ± 0.08^b^	7.98 ± 0.21^b^	9.02 ± 0.10^a^	69.25 ± 2.45^c^	50.70 ± 5.20^a^	92.00 ± 1.42^a^

Note

Data are mean ± *SD* (*n* = 3). Mean values with different superscript letters (a–e) in the same column are significantly different, based on Duncan's multiple‐range test (*p* < 0.05).

#### 3.3.2 Adhesion

The adherence of probiotic strains to epithelial cells is vital for their colonization and survival in the intestinal tract (Morelli, 2007). Additionally, probiotic strains can competitively bind pathogens on epithelial cells and gut‐mucosal binding sites to prevent pathogen attachment (Morrow, Gogineni, & Malesker, 2012). Adhesion to intestinal mucosal cells such as Caco‐2 or HT 29 is commonly used as a prerequisite screening method to assess the adhesion of probiotic strains (Blum et al., 1999). The adhesion of LAB strains to Caco‐2 cells was compared to that of the probiotic strain LGG (Table 3), revealing that *L*.* plantarum* WiKim83, *L*.* plantarum* WiKim84, and *L*.* plantarum* WiKim87 displayed 77.80%, 72.23%, and 73.02% adhesion, respectively, much higher than the 69.33% adhesion observed for LGG (*p* < 0.05). However, the two *P*.* pentosaceus* strains showed lower adhesion than did the three *L*.* plantarum* strains. The adhesion of probiotic strains might be strain‐dependent, and thus, various adhesion properties have been reported according to LAB strain. Argyri et al. (2013) reported that *L*.* plantarum* B282, *L*.* paracasei* E94, and *L*.* pentosus* E108 exhibit high adherence to Caco‐2 cells. Additionally, *L*.* plantarum* Ln4 (89.42%) and *L*.* plantarum* G72 (87.63%), isolated from kimchi, exhibited higher adherence when compared with for *L*.* rhamnosus* (60.12%) (Son et al., 2017). Meanwhile, Oh and Jung (2015) reported the adhesion abilities of seven isolated LAB strains (*P*.* pentosaceus*, *L*.* pentosus*, and *L*.* plantarum*) from fermented millet alcoholic beverage. Among the isolated strains, *P*.* pentosaceus* SW01 showed the highest adhesion of 66.3%, which was lower than that of LGG (74.77%) used as a control. Although in vitro results cannot be directly correlated with in vivo outcomes, a previous study showed a positive association between adherence and intestinal colonization (Wang, Lin, Ng, & Shyu, 2010). Therefore, our results suggested that *L*.* plantarum* WiKim83, *L*.* plantarum* WiKim84, and *L*.* plantarum* WiKim87 strains possess good adhesion characteristics.

#### 3.3.3 Hydrophobicity and auto‐aggregation

An essential characteristic of probiotics is their binding to intestinal mucosa in the gut, which would promote extended retention of the probiotic in intestine and prolonged contact between bacterial and epithelial cells (Gueimonde & Salminen, 2006). Therefore, we evaluated the hydrophobicity and auto‐aggregation of the isolated strains (Table 3). After reacting with chloroform, hydrophobicity ranged from 11.76% to 50.61%, being maximum in *L*.* plantarum* WiKim83 strain (50.61%), similar to that of LGG strains (*p* < 0.05). Auto‐aggregation of the isolated strains was >90% for all strains, except *L*.* plantarum* WiKim86. Although hydrophobicity and auto‐aggregation contribute to bacterial adherence to the intestinal mucosa, they are not absolutely necessary for strong adhesion. A previous study had reported wide variance in strain‐dependent properties within the same bacterial species (Das, Khowala, & Biswas, 2016).

### 3.4 Functional characteristics of LAB

#### 3.4.1 Antimicrobial activity

The isolated strains exhibited antimicrobial activities against different foodborne pathogenic microorganisms (Table 4), with inhibition zones varying in diameter from 7.22 to 17.43 mm. *L*.* plantarum* WiKim83, *L*.* plantarum* WiKim84, and *L*.* plantarum* WiKim87 displayed clear inhibition zones with 10 indicator pathogens, with *L*.* plantarum* WiKim84 exhibiting the highest inhibitory activity against *B*.* cereus*,* S*.* aureus*,* L*.* monocytogenes*, and *S*. Typhimurium (*p* < 0.05). In particular, the antimicrobial activity of isolated LAB strains (*L*.* plantarum* WiKim83, *L*.* plantarum* WiKim84, and *L*.* plantarum* WiKim87) against *B*.* cereus*, *S*.* aureus*,* C*.* perfringens*, *S*. Typhimurium, *E*.* coli*, *V*.* parahaemolyticus*, and *C*.* coli* was higher than that of gentamicin used as a positive control.

**Table 4:** Antimicrobial activity of isolated lactic acid bacteria strains against foodborne pathogens

Indicator microorganism	Inhibition zone (mm)					
	*L*.* plantarum* WiKim83	*L*.* plantarum* WiKim84	*P*.* pentosaceus* WiKim85	*P*.* pentosaceus* WiKim86	*L*.* plantarum* WiKim87	Gentamicin (30 μg/ml)
Gram (+)						
*B*.* cereus*	16.76 ± 0.20^b^	17.43 ± 0.21^a^	15.73 ± 0.16^c^	16.02 ± 0.25^e^	13.94 ± 0.17^c^	15.09 ± 0.31^d^
*S*.* aureus*	14.70 ± 0.28 ^b^	16.36 ± 0.11^a^	13.98 ± 0.06^c^	11.91 ± 0.13^e^	12.45 ± 0.24^d^	7.18 ± 0.01^f^
*L*.* monocytogenes*	8.83 ± 0.21^d^	10.45 ± 0.35^b^	8.49 ± 0.25^e^	7.22 ± 0.06^f^	9.57 ± 0.15^c^	13.23 ± 0.18^a^
*C*.* perfringens*	12.52 ± 0.49^b^	11.59 ± 0.78^c^	10.92 ± 0.86^d^	9.58 ± 0.19^e^	12.92 ± 0.06^a^	n.d.^1^
Gram (−)						
*S*. Typhimurium	14.00 ± 0.11^b^	16.53 ± 0.13^a^	13.35 ± 0.10^c^	11.51 ± 0.16^d^	14.16 ± 0.17^b^	7.87 ± 0.22^e^
*E*.* coli*	13.22 ± 0.28^b^	14.10 ± 0.37^a^	13.90 ± 0.11^a^	12.75 ± 0.10^c^	13.43 ± 0.27^b^	9.68 ± 0.04^d^
*V*.* parahaemolyticus*	13.96 ± 0.60^a^	13.69 ± 0.17^ab^	13.57 ± 0.70^ab^	12.62 ± 0.36^bc^	13.32 ± 0.82^ab^	12.25 ± 0.53^c^
*Y*.* enterocolitica*	10.43 ± 0.08	10.47 ± 0.19	n.d.	n.d.	10.64 ± 0.39	10.49 ± 0.04
*C*.* jejuni*	12.91 ± 0.18^b^	13.00 ± 0.13^b^	12.66 ± 0.18^b^	12.13 ± 0.16^b^	13.02 ± 0.14^b^	18.54 ± 1.41^a^
*C*.* coli*	15.17 ± 0.23^a^	15.27 ± 0.20^a^	14.36 ± 0.13^b^	13.79 ± 0.40^c^	15.53 ± 0.17^a^	13.10 ± 0.45^d^

Note

Data are mean ± *SD* (*n* = 3). Mean values with different superscript letters (a–f) in the same row are significantly different, based on Duncan's multiple‐range test (*p* < 0.05).

^1^ n.d., not detected (inhibition zones with values ≤7 mm were assumed to be devoid of antimicrobial activity).

The production of antimicrobial compounds is an important characteristic for the competitive exclusion of pathogens in the intestines and for probiotic effects (Collado, Gueimonde, Hernandez, Sanz, & Salminen, 2005). The antimicrobial activity of LAB involves various metabolites such as organic acids, bacteriocins, H_2_O_2_, and antimicrobial compounds, thus representing the functional characteristics of probiotics (Crowley, Mahony, & Sinderen, 2013; Kaewnopparat et al., 2013; Li et al., 2014). Lactic acid and other organic acids produced by LAB control the growth of pathogens (Ray & Sandine, 1992). The minimum pH for bacterial growth depends on the strain, and most bacteria are known to grow optimally at pH 6.5 and to be inhibited at pH 4 (Andersen et al., 2009). In this study, consistent with previous reports, the pH of isolated strains decreased from 6.51 to 3.74–3.92 during the growth period, and acidic condition of the culture supernatant affected the growth of pathogens. Many researchers have demonstrated the inhibitory activities of LAB strains against pathogens. Ren et al. (2014) found that fresh overnight cultures of eight *Lactobacillus* strains in human intestines inhibited *E*.* coli*,* B*.* cereus*, and *S*.* aureus*. Previous studies on *L*.* plantarum* have reported that the pathogens *L*.* monocytogenes*,* P*.* aeruginosa*, *S*. Typhimurium, *E*.* coli*,* B*.* cereus*,* Shigella sonnei*, *Enterobacter sakazakii*, and *S*.* aureus* were inhibited by organic acids and exopolysaccharide (EPS) produced by *L*.* plantarum* (Huang et al., 2015; Li et al., 2014). Further, in recent studies, *Salmonella enteric* serovar Typhi growth was shown to be inhibited up to 73% during co‐incubation due to the effects of lactic acid produced by *L*.* lactis* MTCC‐440 (Kumar, Kundu, & Debnath, 2018). *L*.* plantarum* WiKim83, *L*.* plantarum* WiKim84, and *L*.* plantarum* WiKim87 exhibited excellent antimicrobial activities against Gram‐positive and Gram‐negative pathogens and might therefore have beneficial effects when used as a starter culture for food fermentation.

#### 3.4.2 Antioxidant activity

The antioxidant activity of probiotic strains protects the host microflora from radicals during intestinal colonization (Ren et al., 2014) and contributes to the prevention of cardiovascular diseases, gastric ulcers, and diabetes (Kaushik et al., 2009). We evaluated the antioxidant activities of culture supernatants from the isolated strains in terms of their DPPH^+^, ABTS^•+^, and *β*‐carotene activity (Table 5). DPPH^+^ was significantly higher in order of *L*.* plantarum* WiKim83 (35.35%), *L*.* plantarum* WiKim84 (34.05%), and *L*.* plantarum* WiKim87 (34.46%) (*p* < 0.05). The ABTS^•+ ^was also high in *L*.* plantarum* WiKim83 (99.05%) and *L*.* plantarum* WiKim84 (98.90%) (*p* < 0.05). Moreover, *β*‐Carotene activity was highest in *L*.* plantarum* WiKim83 (38.27%) (*p* < 0.05). The isolated LAB strains showed lower DPPH^+^ and *β*‐carotene activity than did the positive control, ascorbic acid, but ABTS^•+ ^was similar between the two. According to a recent study, two *L*.* plantarum* strains showed DPPH^+^activity between 21.08% and 40.97% and *β*‐carotene activity of 31.92%–38.42% (Son et al., 2017). Regarding ABTS^•+^, *P*.* pentosaceus* SC28, isolated from Korean fermented foods, showed activity of 3.34%, whereas *P*.* pentosaceus* R1 and *L*.* plantarum* R6, isolated from sausages, showed activities of 42.4% and 40.1%, respectively (Han, Kong, Chen, Sun, & Zhang, 2017; Son et al., 2018). Although the mechanism of antioxidant activity in LAB has not yet been clarified, it is known to be related to metabolites such as SOD, NADH oxidase, and various antioxidant enzymes produced by such strains (Arasu et al., 2014; Lin & Chang, 2000; Son et al., 2018). In addition, antioxidant activities of EPS such as glucans, fructans, and gluco‐ and fructo‐oligosaccharides have been reported in some LAB (Kodali & Sen, 2008; ; Liu, Tseng, et al., 2011). Among the antioxidant activities of the five LAB strains, the ABTS^•+^ of the isolated strains was superior to that reported previously. Especially, *L*.* plantarum* WiKim83 showed the highest activity based on all three antioxidant activity indices, suggesting that it could have potential benefits as a health food for antioxidation.

**Table 5:** Antioxidant activity of the culture supernatants from isolated lactic acid bacteria strains

	Antioxidant activity (%)					
	*L*.* plantarum* WiKim83	*L*.* plantarum* WiKim84	*P*.* pentosaceus* WiKim85	*P*.* pentosaceus* WiKim86	*L*.* plantarum* WiKim87	Ascorbic acid (1 mg/ml)
DPPH^+^	35.35 ± 3.98^b^	34.05 ± 1.70^b^	32.19 ± 1.14^bc^	29.33 ± 1.60^c^	34.46 ± 0.92^b^	99.99 ± 0.10^a^
ABTS^•+^	99.05 ± 0.90^ab^	98.90 ± 0.10^ab^	98.85 ± 0.28^b^	97.69 ± 0.60^c^	97.41 ± 0.94^c^	99.99 ± 0.01^a^
*β*‐Carotene	38.27 ± 1.10^b^	33.37 ± 0.93^cd^	31.23 ± 1.07^d^	24.32 ± 0.99^e^	35.36 ± 1.62^c^	93.33 ± 0.49^a^

Note

Data are mean ± *SD* (*n* = 3). Means with different superscript letters (a–e) in the same row are significantly different, based on Duncan's multiple‐range test (*p* < 0.05).

#### 3.4.3 *β*‐Galactosidase activity

**Figure 1:**
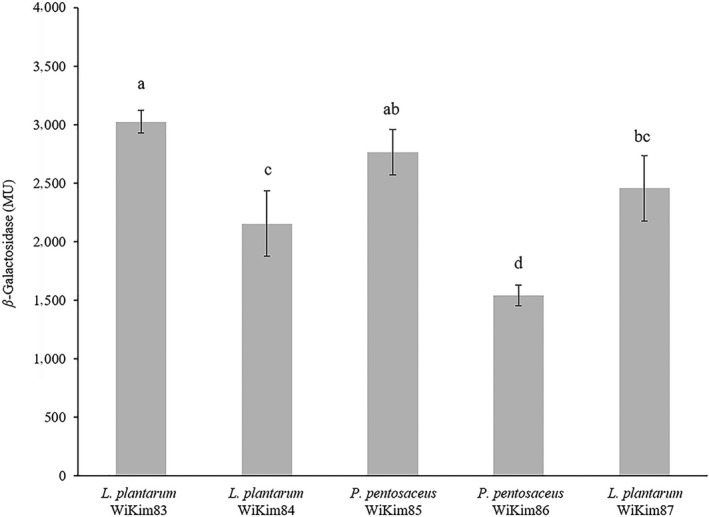
*β*‐Galactosidase activity of isolated lactic acid bacteria strains. The activities are expressed in Miller units. Error bars indicate the *SD* of three independent experiments. Means in each column, having a common letter, are significantly different from the others (*p* < 0.05)

*β*‐Galactosidase possesses the important characteristics of hydrolysis and the transglycosylation of lactose (Bras, Fernandes, & Ramos, 2010). LAB strains with *β*‐galactosidase activity can convert lactose into useful short‐chain fatty acids and are widely used for lactose intolerance treatment and in the dairy industry (Naidu, Bidlack, & Clemens, 1999). *β*‐Galactosidase activity among the five LAB strains was the highest in *L*.* plantarum* WiKim83 (3,023 MU), followed by *L*.* plantarum* WiKim85 (2,763 MU) and *L*.* plantarum* WiKim87 (2,457 MU) (Figure 1; *p* < 0.05). Previous studies have reported the *β*‐galactosidase activity of LAB, and two *L*.* plantarum* strains showed 1,300–3,300 MU, whereas six strains of *P*.* pentosaceus* had values of 114–5,990 MU (Son et al., 2017; Vidhyasagar & Jeevaratnam, 2013). In particular, among LAB strains, the *β*‐galactosidase activity of lactobacilli including *Lactobacillus reuteri*, *Lactobacillus fermentum*, *Lactobacillus crispatus*, and *L*.* plantarum* has been characterized (Kim & Rajagopal, 2000; Liu, Kong, et al., 2011; Son et al., 2017; Splechtna et al., 2006). The basic function of the starter culture is to convert the milk sugar lactose into an acid product and contribute to the preservation, digestibility, texture, and flavor of the fermented product (Hébert, Raya, Tailliez, & Giori, 2000; Husain, 2010). In this study, *β*‐galactosidase activity was significantly different across the LAB strains. Especially, *L*.* plantarum* WiKim83 showed high enzyme activity, which is highly useful for the dairy industry.

### 3.5 Multivariate analysis of functional LAB characteristics

**Figure 2:**
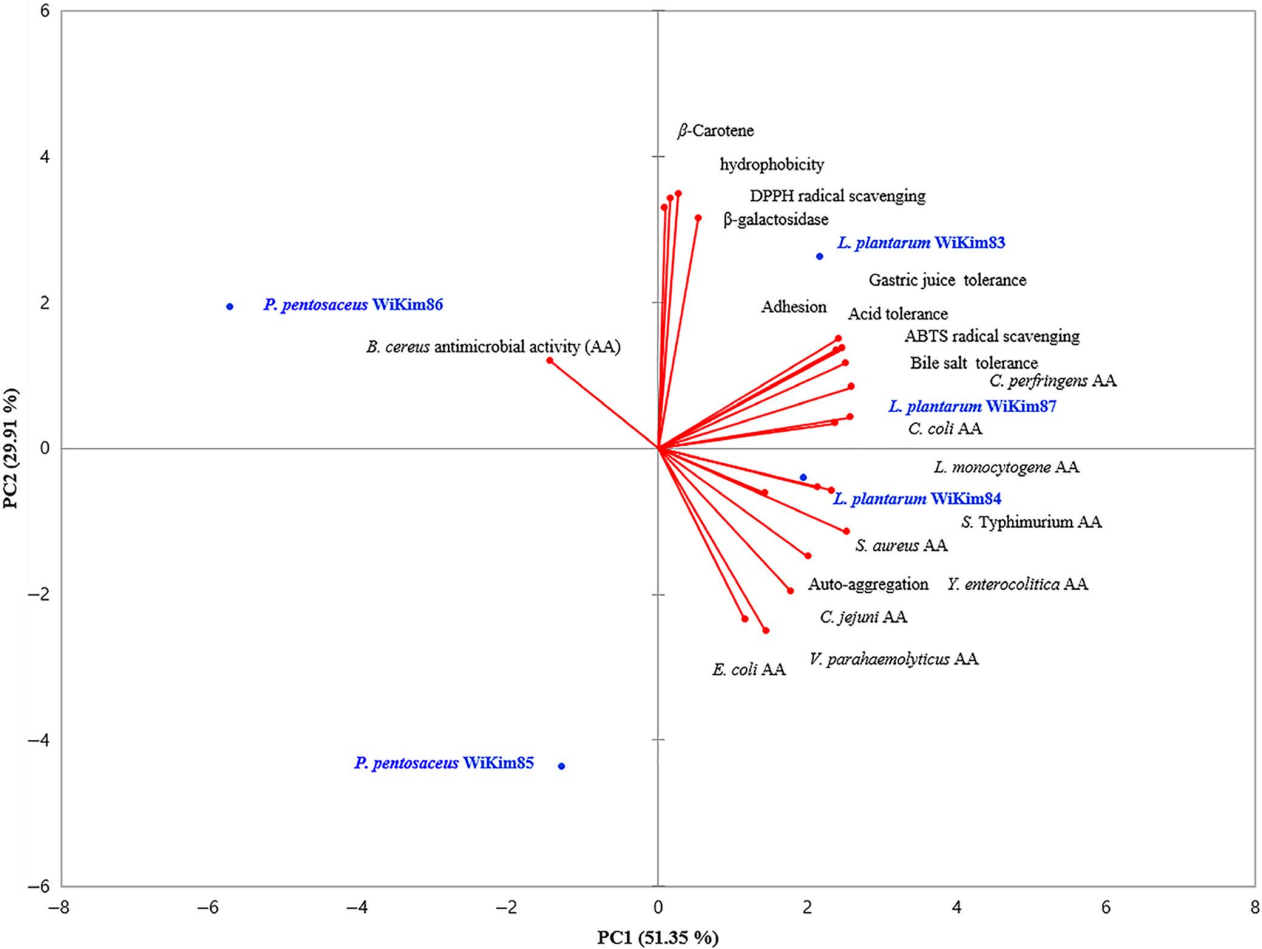
Principal component analysis (PCA) biplot based on the probiotic properties of isolated lactic acid bacteria strains. PCA results show that 81.26% of the total variation was distributed in PC1 (51.35%) and PC2 (29.91%)

As the final step in the selection of new functional LAB with probiotic potential, the main features of each LAB were highlighted through PCA and the most promising strains were selected (Figure 2). PCA results showed that the first and second factors accounted for 81.26% of the total variance. The first factor (PC1), which accounted for 51.35% of the total variance, had the highest eigenvalue of 10.27, with total variance of the second factor (PC2) being 29.91%, with an eigenvalue of 5.98. PC1 was highly influenced by probiotic properties such as GIT tolerance and adherence, whereas PC2 was affected by functional properties such as antioxidant, enzymatic, and antimicrobial activity. PC1 was strongly influenced by probiotic properties such as GIT resistance and adhesion, and PC2 was affected by functional properties such as antioxidant, *β*‐galactosidase, and antimicrobial activities. *L*.* plantarum* WiKim84 of the PC1‐positive side and PC2‐negative side was characterized by its antimicrobial activity. *L*.* plantarum* WiKim83 and *L*.* plantarum* WiKim87, located on positive sides of PC1 and PC2, were selected as the most promising strains with probiotic properties including antioxidant and *β*‐galactosidase activities.

## 4 CONCLUSIONS

This study evaluated and selected promising multifunctional LAB strains for use in starter culture for the fermentation of food. The LAB were isolated from kimchi, evaluated for probiotic properties relative to those of commercial probiotic strains, and selected through statistical analysis of their functionality, such as antimicrobial activity, antioxidant activity, and *β*‐galactosidase activity. Through this process, two strains, *L*.* plantarum* WiKim83 and *L*.* plantarum* WiKim87, were finally selected as potential candidates. *L*.* plantarum* WiKim83 and *L*.* plantarum* WiKim87 have high GIT tolerance, adhesion, antimicrobial activities against food pathogens, and antioxidant and *β*‐galactosidase activities, due to which they were considered to be the most applicable strains. However, further studies are required to assess the strains in vivo and identify the technical characteristics in future.

## CONFLICT OF INTEREST

The authors declare that they do not have any conflict of interest.

## ETHICAL REVIEW

This study does not involve any human or animal testing.

## INFORMED CONSENT

Not applicable.
