# Supplementary material for: Multifunctional properties of Lactobacillus plantarum strains WiKim83 and WiKim87 as a starter culture for fermented food
Source: Food Sci Nutr. 2019 Jul 2;7(8):2505–16. doi: 10.1002/fsn3.1075 (PMC6694436; doi:10.1002/fsn3.1075)
Supplement: Supplementary file 1 [file FSN3-7-2505-s001.docx]

Supplementary table 1. Antimicrobial activity of isolated lactic acid bacteria strains against foodborne pathogens

| Strains | Inhibition zone (mm) | | |
| --- | --- | --- | --- |
|  | *S. aureus* | *L. monocytogenes* | *E. coli* |
| WiKim3-1 | 19.00 ± 0.20 ^f^ | 10.04 ± 0.15 ^bcd^ | 8.15 ± 0.06 ^c^ |
| WiKim5-1 | 23.23 ± 0.17 ^d^ | 10.47 ± 0.44 ^ab^ | 8.16 ± 0.19 ^d^ |
| WiKim84 | 24.50 ± 0.54 ^bc^ | 10.80 ± 0.23 ^a^ | 9.03 ± 0.16 ^b^ |
| WiKim85 | 27.23 ± 0.74 ^a^ | 10.13 ± 0.36 ^bc^ | 8.65 ± 0.18 ^d^ |
| WiKim83 | 25.50 ± 0.62 ^b^ | 10.93 ± 0.08 ^a^ | 9.74 ± 0.22 ^a^ |
| WiKim87 | 23.63 ± 0.64 ^cd^ | 10.70 ± 0.16 ^a^ | 9.54 ± 0.45 ^a^ |
| WiKim5-2 | 21.15 ± 0.16 ^e^ | 9.62 ± 0.22 ^d^ | 8.09 ± 0.06 ^e^ |
| WiKim1-1 | 21.97 ± 0.76 ^e^ | 9.66 ± 0.19 ^d^ | 8.25 ± 0.14 ^d^ |
| WiKim86 | 25.51 ± 0.96 ^b^ | 10.07 ± 0.08 ^bcd^ | 8.26 ± 0.50 ^d^ |
| WiKim5-4 | 21.51 ± 0.55 ^e^ | 9.86 ± 0.32 ^cd^ | 8.25 ± 0.19 ^d^ |

Data are mean ± SD (*n* = 3).

^a–f^ Means with different letters in the same column are significantly different, based on Duncan’s multiple-range test (*P* < 0.05).
